# *APOE* *ε*4 carriage associates with improved myocardial performance from adolescence to older age

**DOI:** 10.1186/s12872-024-03808-z

**Published:** 2024-03-21

**Authors:** Constantin-Cristian Topriceanu, Mit Shah, Matthew Webber, Fiona Chan, Hunain Shiwani, Marcus Richards, Jonathan Schott, Nishi Chaturvedi, James C. Moon, Alun D. Hughes, Aroon D. Hingorani, Declan P. O’Regan, Gabriella Captur

**Affiliations:** 1 UCL MRC Unit for Lifelong Health and Ageing, University College London, London, UK; 2 UCL Institute of Cardiovascular Science, University College London, London, UK; 3 Cardiac MRI Unit, Barts Heart Centre, London, UK; 4 Imperial Centre for Translational and Experimental Medicine, National Heart and Lung Institute, Imperial College London, London, UK; 5 MRC London Institute of Medical Science, Imperial College London, London, UK; 6 Dementia Research Centre, UCL Queen Square Institute of Neurology, London, UK; 7 BHF Research Accelerator, University College London, London, UK; 8 Health Data Research, University College London, London, UK; 9 Cardiology Department, Centre for Inherited Heart Muscle Conditions, The Royal Free Hospital, Pond Street, Hampstead, London, UK

**Keywords:** Apolipoprotein *ε*4, Cardiovascular disease, Myocardial contraction fraction

## Abstract

**Background:**

Although *APOE ε*4 allele carriage confers a risk for coronary artery disease, its persistence in humans might be explained by certain survival advantages (antagonistic pleiotropy).

**Methods:**

Combining data from ~ 37,000 persons from three older age British cohorts (1946 National Survey of Health and Development [NSHD], Southall and Brent Revised [SABRE], and UK Biobank) and one younger age cohort (Avon Longitudinal Study of Parents and Children [ALSPAC]), we explored whether *APOE ε*4 carriage associates with beneficial or unfavorable left ventricular (LV) structural and functional metrics by echocardiography and cardiovascular magnetic resonance (CMR).

**Results:**

Compared to the non-*APOE ε*4 group, *APOE ε*4 carriers had similar cardiac phenotypes in terms of LV ejection fraction, E/e’, posterior wall and interventricular septal thickness, and LV mass. However, they had improved myocardial performance resulting in greater LV stroke volume generation per 1 mL of myocardium (higher myocardial contraction fraction). In NSHD (*n* = 1467) and SABRE (*n* = 1187), *ε*4 carriers had a 4% higher MCF (95% CI 1–7%, *p* = 0.016) using echocardiography. Using CMR data, in UK Biobank (*n* = 32,972), *ε*4 carriers had a 1% higher MCF 95% (CI 0–1%, *p* = 0.020) with a dose-response relationship based on the number of *ε*4 alleles. In addition, UK Biobank *ε*4 carriers also had more favorable radial and longitudinal strain rates compared to non *APOE ε*4 carriers. In ALSPAC (*n* = 1397), *APOE ε*4 carriers aged < 24 years had a 2% higher MCF (95% CI 0–5%, *p* = 0.059).

**Conclusions:**

By triangulating results in four independent cohorts, across imaging modalities (echocardiography and CMR), and in ~ 37,000 individuals, our results point towards an association between *ε4* carriage and improved cardiac performance in terms of LV MCF. This potentially favorable cardiac phenotype adds to the growing number of reported survival advantages attributed to the pleiotropic effects *APOE* ε4 carriage that might collectively explain its persistence in human populations.

**Supplementary Information:**

The online version contains supplementary material available at 10.1186/s12872-024-03808-z.

## Introduction

*Apolipoprotein ε* (*APOE ε*) mediates the binding of low-density lipoprotein (LDL) to peripheral receptors. Given the existence of two single-nucleotide polymorphisms, namely rs429358 and rs7412, there are three *APOE ε* isoforms coded by the alleles *ε*2, *ε*3 and *ε*4 giving rise to six genotypes namely ε2ε2, ε2ε3, ε2ε4, ε3ε3, ε3ε4 and ε4ε4 with the commonest being ε3ε3 [1].

*Apolipoprotein ε*4 is regarded to be a major risk factor for developing Alzheimer’s disease [2] even from young age (especially in females [3]) and with a clear dosage effect (carriage of two *ε*4 alleles are associated with a higher risk than 1). In addition, it may associate with decreased physical performance in older age [4] and decrease cognitive performance (e.g., verbal episodic memory) in healthy young adults [5]. Yet despite its adverse associations, this ancestral allele has persisted in human populations instead of being replaced by the more recently evolved alleles, *ε*3 and *ε*2 [6] suggesting its carriage might be conferring some survival advantages. Indeed, *APOE ε*4 carriers have been shown to have increased fertility [7, 8], resistance to infections [7], decreased perinatal and infant mortality [7], decreased chronic airway obstruction [9], fewer arterial aneurysms [9] and peptic ulcers [9], less liver disease and slight cognitive advantages [7, 10].

In terms of the cardiovascular system, carriage of *ε*4 (rs429358-cytosine and rs7412-cytosine) has been associated with adverse clinical sequelae including ischaemic heart disease (IHD) [11], hypertension [12], diabetes [13] and high LDL [14]. Moreover, heart function was also suggested to be a mediator in the association between *APOE ε*4 and gray matter decline [15]. However, these findings were inconsistent and not reproducible enough to support a causal role of *APOE ε*4 in CVD and its risk factors.

To date it remains unclear whether *APOE ε*4 carriage independently associates with a better or worse long-term cardiac phenotype in terms of heart size and function. Using cohort data from the Avon Longitudinal Study of Parents and Children (ALSPAC), Medical Research Council (MRC) 1946 National Survey of Health and Development (NSHD), Southall And Brent Revised (SABRE) and United Kingdom (UK) Biobank, we explored this association.

## Methods

### Study population

The ALSPAC is a birth cohort that recruited 14,541 pregnant women with an expected date of delivery in 1991–1992 [16].

The MRC NSHD is the world’s longest-running birth cohort with continuous follow-up. In 1946 in Britain, 5362 individuals (2547 males and 2815 females) born in the same week in March were enrolled. Participants were invited for periodic follow-ups in which health and socio-economic assessments were performed which have been described elsewhere [17].

The SABRE study is a tri-ethnic cohort of European, South Asian, and African Caribbean participants living in North and West London. Between 1988 and 1981, participants aged 40–69 years were randomly selected from 5-year age and sex stratified primary care lists (*n* = 4063) and workplaces (*n* = 795). Full details have been described elsewhere [18].

The UK Biobank is a large prospective cohort study with more than half a million individuals recruited between 2006 and 2010 when study participants were aged 40–69 years old, and features demographic, genetic, health outcome and imaging data for participants [19]. Details of subjects’ comorbidities were obtained through self-reported diagnoses and International Classification of Disease (ICD-9 and ICD-10) codes from linked medical records This project was conducted using the UK Biobank (UKBB) resource under application numbers 40,616 and 46,696.

All ALSPAC, NSHD, SABRE and UKBB participants from whom the *APOE ε* genotype was known and had structural cardiac imaging were included in this study.

### Outcomes: echocardiographic data

In ALSPAC, echocardiography was performed when study participants were 17 and 24 years by 1–2 experienced echocardiographers in accordance with the American Society of Echocardiography (ASE) guidelines with good reproducibility (both intraobserver and interobserver correlation coefficients ranged between 0.75 to 0.93 [20]). Since non-attendance to clinic visits is especially relevant within this group [21], echocardiography measurements were either averaged if more than one scan was available, or the one available scan was used to reduce the bias associated with data missingness.

In NSHD, when study members were 60–64 years (2006–2010), British-based NSHD participants who had not been lost to follow-up or withdrawn, were invited to attend a clinic-based assessment that included resting transthoracic echocardiography using General Electric (GE) Vivid I machines. The echocardiographic protocol included long and short axis (LAX and SAX), apical 5-, 4-, 3- and 2- chamber, and aortic SAX views [17]. In NSHD, echocardiography quality assurance was evaluated based on blind duplicate readings showing excellent inter- and intrareader variability (coefficients > 0.80) [22].

In SABRE, study members were invited between 2008 and 2012 to a clinic visit in which echocardiographic data was acquired using a Phillips iE33 ultrasound machine S5–1 phased array and a X3–1 matrix transducer and analyzed in line with the with the ASE guidelines. For structural and volumetric metrics, the inter- and intra-observer agreement was also high in SABRE (coefficients > 0.71) [23].

In all three cohorts, echocardiographic data provided left ventricular (LV) ejection fraction (EF), E/e’, systolic and diastolic LV posterior wall and interventricular septal thickness (LVPWTs/d, IVSs/d), LV mass (LVmass) and the stroke volume (SV). Myocardial contraction fraction (MCF) was calculated as the ratio between stroke volume and myocardial volume. Although indexation to body surface area (BSA), is commonly done in clinical practice, BSA is a poor indexation metric as it creates a bias for overweight individuals [24]. Although indexation to allometric height is a better alternative [24], indexation might lead to spurious associations, as the exposure might be associated with height/weight rather than with the outcome itself. Therefore, we used unindexed echocardiographic outcomes in all subsequent analyses.

### Outcomes: cardiovascular magnetic resonance data

Participants in the UK Biobank were randomly invited for a CMR scan on a 1.5 T Siemens Aera scanner from 2014. Briefly, the CMR imaging protocol consisted of three long-axis views and a complete short axis stack of balanced steady state free precession cines [25]. Grey-scale short axis cine stacks were automatically segmented using a deep learning neural network that has optimised for UKBB scan images, with human expert level performance [26]. The short-axis segmentations underwent post-processing to compute end-systolic, end-diastolic and stroke volumes in both ventricles [27]. Left ventricular mass (LVM) was computed from left ventricular volume (assuming a density of 1.05 g/ml). Left ventricular wall thickness was computed as the perpendicular radial-line distance between endocardial and epicardial surfaces at end-diastole for each of the 17 myocardial segments as defined by the American Heart Association (AHA) [28]. MCF was derived as above. Thickness of the IVS was calculated as the mean wall thickness of segments 2, 3, 8, 9 and 14, while PWT was taken as the mean of segments 5,6, 11, 12, and 16. To compute longitudinal and radial peak diastolic strain rates, non-rigid image co-registration was performed between successive frames to enable dynamic motion tracking of the heart during the cardiac cycle [29]. Unindexed CMR metrics were used in all subsequent analyses as discussed above.

### Exposures: *APOE ε* genotype

In ALSPAC, genetic samples were available for 2009 children. *APOE ε* genotype was appraised using integrated single-label liquid phase assay in 2011 [30].

In NSHD, blood samples were collected at age 53 by a trained research nurse, and DNA was extracted [31]. Genetic analysis of stored samples took place in in 1999 and 2006–2010. In SABRE, blood samples were collected during baseline studies in 1988–1991 and during follow-up from 2007 to 2012 [18]. Genotyping of rs439358 and rs7412 was conducted at the Exeter University for SABRE and by LGC, Huddleston, UK for NSHD [32].

Genotyping of UK Biobank participants is detailed elsewhere [33], however in brief, genotyping for 488,252 subjects was performed using the UK BiLEVE or UK Biobank Axiom arrays and imputation based on the HaplotypeReference Consortium and UK10K + 1000 Genomes panels. Imputation V3 (in GRCh37 coordinates) was used for the current study. Genotypes in their released PLINK-format files were used on the DNANexus platform (https://www.dnanexus.com/).

Based on the presence or absence of *APOE ε4*, genotypes were categorically defined as: non-*APOE ε*4 carriers (*ε*2*ε*2, *ε*2*ε*3, *ε*3*ε*3), heterozygous-*APOE ε*4 (ε2ε4 and ε3ε4) or homozygous-*APOE ε*4 (*ε*4*ε*4). Heterozygous-*APOE ε*4 and homozygous-*APOE ε*4 were further grouped into *APOE ε*4 carriers.

### Covariates

Sex was recorded as male or female. The age, weight, and height at the time of the imaging were used to compute the body mass index (BMI) in all 3 cohorts. In NSHD, participants’ socioeconomic position (SEP) was evaluated at the time of echocardiography according to UK Surveys Registrar General’s social class, dichotomized as manual or non-manual. In ALSPAC, father’s SEP was available in the same format as the NSHD. In UK Biobank, we used the Townsend deprivation index scores derived from national data about ownership and unemployment aggregated by postcodes [34]. The presence of cardiovascular disease (CVD), diabetes or high cholesterol was recorded as 1 = present or 0 = absent. In ALSPAC, congenital heart disease was used instead of CVD. Congenital heart disease and CVD were self-reported or GP-based diagnoses, while diabetes was defined based on doctor diagnosis and the use of diabetes medications. High cholesterol was defined based on the use of lipid-lowering drugs or as a total cholesterol higher than 240 mg/dl.

### Statistics

All analyses were performed in R 4.0 [35]. For all analyses, a two-tailed *p*-value < 0.05 was considered statistically significant.

Distribution of data were assessed on histograms and using Shapiro-Wilk test. Continuous variables are expressed as mean ± 1 standard deviation (SD) or median (interquartile range) as appropriate; categorical variables, as counts and percent.

**Fig. 1:**
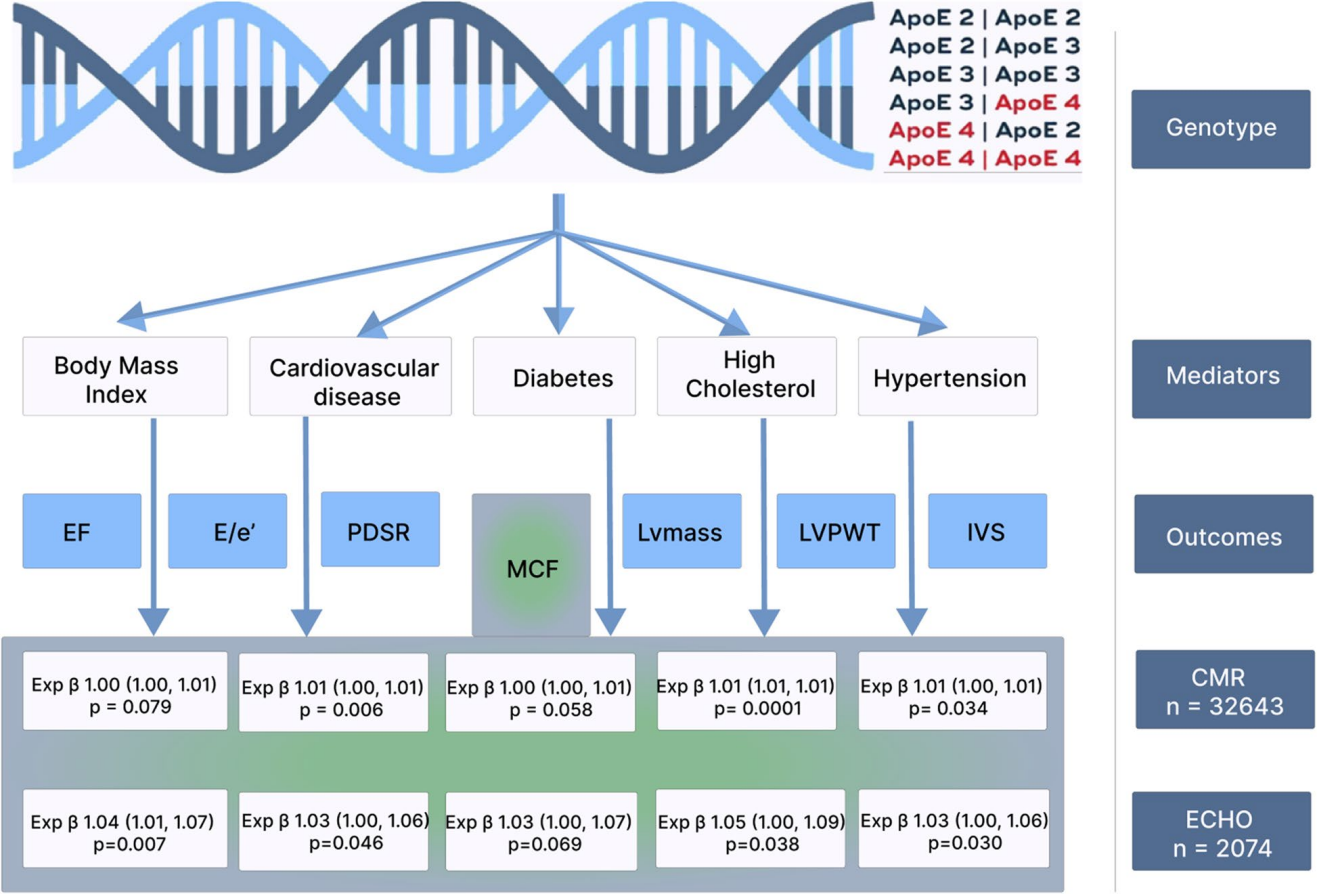
Associations between *APOE ε4* genotypes and echocardiographic and cardiac MRI data in older age. As *APOE ε4* carriers had a higher myocardial contraction fraction, the mechanistic pathways were explored by adjusting the models for mediators (body mass index, cardiovascular disease, diabetes, high cholesterol, and hypertension). EF, ejection fraction; IVS, interventricular septal thickness; LVmass, left ventricular mass, LVPW left ventricular posterior wall thickness; MCF myocardial contraction fraction; PDSR, longitudinal/radial peak diastolic strain rate

In the main analysis, we compared non-*APOE ε*4 carriers with *APOE ε*4 carriers. Given the skewed distributions of echocardiographic and CMR data, generalized linear models with gamma distribution and log link were used to investigate the association of *APOE ε4* genotypes as the exposures to predict the continuous echocardiographic and CMR variables as the outcomes. As the longitudinal and radial PDSR also spanned negative values, generalized linear models with Gaussian distribution and identity link were used instead. Being a combination of gene variants, *APOE ε* genotype is expected to be an instrumental variable and therefore unconfounded. Thus, Model 1 was unadjusted. To obtain more precise regression estimates, Model 2 was adjusted for factors associated with the outcome, namely age, sex, and SEP. To explore the mechanistic pathway downstream of *APOE ε* genotype but upstream of the echocardiographic outcomes, subsequent models were adjusted for mediators as follows: Model 3 for BMI; Model 4 for the presence of CVD; Model 5 for diabetes; Model 6 for high cholesterol; and Model 7 for hypertension (Fig. 1). Model assumptions were verified with regression diagnostics and found to be satisfied.

For all the models, regression estimates were obtained separately for ALSPAC, NSHD, SABRE and UK Biobank (i.e., cohort specific analyses). Since both NSHD and SABRE participants had echocardiography and were of a similar age (i.e., > 60 years on average), random-effects meta-analyses were performed across these 2 cohorts. Heterogeneity was evaluated using the Cochran Q test and Higgins I^2^ statistic. Although ALSPAC had echocardiographic data, participants were < 24 years of age and thus were not included in the meta-analysis given the heterogeneity with the older cohorts. Since UK Biobank had CMR data, it was not included in the meta-analysis.

To explore dose responses, *APOE ε4* genotypes were recoded as an ordered category based on the number of *ε4* possessed. Thus, class 0 = *ε2ε2, ε2ε3, ε2ε3*; class 1 = *ε2ε4* and *ε3 ε4*; and class 2 = *ε4ε4*. Given the existence of 3 classes, generalized linear models with gamma distribution (or Gaussian distribution for longitudinal and radial PDSR) and orthogonal polynomial contrasts with 2 equally spaced levels (i.e., linear and quadratic) were employed to look for a dose response by *ε4* variants. Then, we filtered significant results correcting for multiple testing at a false discovery rate (FDR) of 0.15.

As a sensitivity analyses, *APOE ε*4 carriers were split into heterozygous-*APOE ε*4 (*ε*2*ε*4 and *ε*3*ε*4) and homozygous-*APOE ε*4 (*ε*4*ε*4), and all the analyses were replicated as above.

As an extra sensitivity analysis, we explored the association between *APOE ε*4 carriage and stroke volume.

## Results

### Participant characteristics

Participants with available *APOE ε*4 genotype and at least one cardiac imaging metric were included, yielding a total of 37,023 participants (*n* = 1397 from ALSPAC, *n* = 1467 from NSHD, *n* = 1187 from SABRE and *n* = 32,972 from UK Biobank). Their characteristics are shown in Table 1. In total, there were 843 homozygous-*APOE ε*4 and 9460 heterozygous-*APOE ε*4 individuals, with a similar prevalence across ALSPAC, NSHD, SABRE, and UK Biobank. ALSPAC participants were younger (< 24 years), had a lower BMI (median 23), and were less likely to have diabetes, cardiovascular diseases, high cholesterol, or hypertension (< 5%). SABRE participants were more likely to be males (76.75%), have a higher BMI (median 27 years) or suffer from hypertension (58.98%) compared to NSHD and UK Biobank participants. On the other hand, UK Biobank participants were least likely to suffer from CVD (6.53%), diabetes (18.64%), or hypertension (27.62%).

**Table 1:** General characteristics of study participants

		NSHD	SABRE	UK Biobank	ALSPAC
Variable		Count (%), ***n*** = 1467	Count (%), ***n*** = 1187	Cohort (%), ***n*** = 32,972	Cohort (%), ***n*** = 1397
**Exposure:** ***APOE*** *ε4* **genotype**	*ε*2*ε*2	8 (0.55%)	6 (0.51%)	178 (0.54%)	12 (0.86%)
	*ε*2*ε*3	169 (11.54%)	130 (11.95%)	4046 (12.27%)	200 (14.32%)
	*ε*2*ε*4	44 (3.00%)	27 (2.28%)	773 (2.34%)	35 (2.50%)
	*ε*3*ε*3	855 (57.36%)	726 (61.16%)	19,587 (59.41%)	801 (57.34%)
	*ε*3*ε*4	343 (23.41%)	269 (22.66%)	7647 (23.19%)	322 (23.05%)
	*ε*4*ε*4	46 (3.14%)	29 (2.44%)	741 (2.25%)	27 (1.93%)
**Echo at 60–64 years**	**APOE** ***ε4*** **status**	**Median (IQR)**	**Median (IQR)**	**Median (IQR)**	**Median (IQR)**
EF	−/−	65.06 (60.02, 69.27)	62.17 (55.81, 68.51)	59.75 (55.84, 63.69)	65.77 (61.67, 69.29)
	+/−	64.73 (59.33, 69.43)	63.05 (57.66, 69.74)	59.64 (55.85, 63/66)	65.61 (60.90, 69.47)
	+/+	66.68 (61.94, 69.34)	62.07 (56.71, 67.73)	60.10 (56.37, 63.95)	65.26 (62.57, 67.21)
E/e’	−/−	7.72 (6.51, 9.20)	8.12 (7.11, 10.78)	N/A	5.42 (4.69, 6.20)
	+/−	7.52 (6.30, 8.87)	8.91 (7.51, 10.53)	N/A	5.43 (4.71, 6.19)
	+/+	7.18 (6.07, 8.37)	8.35 (6.73, 9.86)	N/A	5.16 (4.47, 5.55)
L_PDSR_	−/−	N/A	N/A	1.59 (1.23, 2.00)	N/A
	+/−	N/A	N/A	1.61 (1.25, 2.01)	N/A
	+/+	N/A	N/A	1.62 (1.27, 2.00)	N/A
R_PDSR_	−/−	N/A	N/A	−5.70 (−7.03, −4.38)	N/A
	+/−	N/A	N/A	−5.77 (−7.05, −4.43)	N/A
	+/+	N/A	N/A	−5.71 (−7.05, − 4.43)	N/A
LVmass	−/−	108.89 (92.86, 131.80)	93.38 (79.59, 107.72)	82.71 (68.41, 100.79)	123.90 (105.44, 150.22)
	+/−	108.38 (87.62, 137.70)	93.75 (80.83, 109.64)	82.53 (68.51, 100.49)	123.4 (102.7, 148.7)
	+/+	113.25 (98.08, 127.13)	91.24 (80.83, 109.2)	81.69 (68.78, 100.88)	132.65 (015.95, 162.72)
MCF	−/−	0.47 (0.37, 0.59)	0.58 (0.49, 0.70)	1.07 (0.95, 1.21)	0.45 (0.39, 0.51)
	+/−	0.51 (0.39, 0.65)	0.60 (0.50, 0.71)	1.08 (0.95, 1.22)	0.46 (0.40,0.52)
	+/+	0.53 (0.42, 0.60)	0.62 (0.55, 0.68)	1.09 (0.97, 1.23)	0.45 (0.38, 0.50)
LVPWT_s_	−/−	1.57 (1.40, 1.74)	1.48 (1.35, 1.62)	N/A	1.32 (1.23, 1.44)
	+/−	1.58 (1.42, 1.80)	1.45 (1.32, 1.59)	N/A	1.32 (1.22, 1.43)
	+/+	1.60 (1.47, 1.74)	1.39 (1.26, 1.60)	N/A	1.33 (1.24, 1.48)
LVPWT_d_	−/−	0.98 (0.87, 1.09)	1.02 (0.92, 1.13)	5.65 (5.14, 6.21)	0.86 (0.79, 0.95)
	+/−	0.98 (0.88, 1.10)	1.01 (0.91, 1.12)	5.64 (5.15, 6.23)	0.85 (0.78, 0.93)
	+/+	0.96 (0.87, 1.04)	0.98 (0.90, 1.10)	5.67 (5.17, 6.26)	0.85 (0.78, 0.94)
IVS_s_	−/−	1.50 (1.34, 1.68)	1.58 (1.42, 1.74)	N/A	1.17 (1.07, 1.30)
	+/−	1.51 (1.35, 1.69)	1.57 (1.40, 1.76)	N/A	1.17 (1.07, 1.28)
	+/+	1.50 (1.36, 1.64)	1.51 (1.44, 1.70)	N/A	1.15 (1.04, 1.29)
IVS_d_	−/−	1.04 (0.91, 1.18)	1.15 (1.03, 1.30)	5.59 (4.98, 6.13)	0.83 (0.75, 0.92)
	+/−	1.04 (0.90, 1.18)	1.14 (1.01, 1.29)	5.58 (4.98, 6.12)	0.82 (0.74, 0.90)
	+/+	1.09 (0.93, 1.15)	1.09 (1.04, 1.21)	5.58 (4.97, 6.12)	0.84 (0.76, 0.92)
**Covariates**		**Count (%) or Median (IQR)**	**Count (%) or Median (IQR)**	**Count (%) or Median (IQR)**	**Count (%) or Median (IQR)**
Age		62 (0)	52.08 (7.27)	63.63 (7.57)	20.5 (0)
Sex, male		708 (48.32%)	911 (76.75%)	15,750 (47.77%)	581 (41.59%)
BMI		26.94 (24.49, 30.22)	27.00 (24.35, 29.90)	25.84 (23.46, 28.77)	22.96 (20.73, 25.60)
CVD, Yes		875 (8.72%)	232 (19.55%)	2153 (6.53%)	0 (0%)
Diabetes, Yes		321 (21.88%)	256 (21.57%)	1991 (6.04%)	8 (0.57%)
High cholesterol, Yes		282 (19.22%)	235 (19.80%)	6145 (18.64%)	60 (5.16%)
Hypertension		719 (50.65%)	700 (58.98%)	9106 (27.62%)	64 (5.72%)

Participants were included in the study if they had the apolipoprotein *APOE ε* genotype and at least one echocardiographic parameter available

*−/−, no APOE ε4 carriage; +/−, heterozygous APOE ε4 carriage; +/+, homozygous; ALSPPAC, Avon Longitudinal Study of Parents and Children; APOE ε4 carriage; APOE, apolipoprotein E, BMI, body mass index; CVD, cardiovascular disease; Echo, echocardiography; EF, ejection fraction; IQR, interquartile, IVSs*_*/d*_*, interventricular septal thickness in systole/diastole; LVmassi, left ventricular mass indexed to body surface area, LVPWT*_*s/d*_ *left ventricular posterior wall thickness in systole/diastole; MCF*_*i*_*, myocardial contraction fraction; N/A, not applicable; NSHD, National Survey of Health and Development; L/R*_*PDSR*_*, longitudinal/radial peak diastolic strain rate; SABRE, Southall and Brent Revisited*

### Associations between *APOE ε4* genotypes and echocardiographic data

In NSHD, when compared to the non-*APOE ε*4 group, *APOE ε*4 carriers had a 6% higher LV MCF (95% confidence interval [CI] 0–12%, *p* = 0.050) which persisted unattenuated after adjusting for sex and SEP (*p* = 0.038) and diabetes (*p* = 0.056), was attenuated to 5% after adjusting for BMI (95% CI 0–11%, *p* = 0.064), CVD (95% CI 0–12%, *p* = 0.112) or hypertension (95% CI 1–11%, *p* = 0.081), and increased to 8% after adjusting for high cholesterol (95% CI 1–14%, *p* = 0.020, Supplementary Table S1). Similarly, *APOE ε*4 carriers had a 5% higher LVmass *p* = 0.057 which was increased to 6% after adjusting for CVD (*p* = 0.040) or hypertension (*p* = 0.040), and to 7% after adjusting for diabetes (*p* = 0.024). No significant associations were found in SABRE (Supplementary Table S2). Moreover, in NSHD *APOE ε*4 carriers had an 8% higher SV 95%CI 3–12% *p* = 0.001 (Supplementary Table S5).

In the NSHD + SABRE meta-analyses, compared to the non-*APOE ε*4 group, *APOE ε*4 carriers had similar cardiac phenotypes in terms of EF, E/e’, LVPWT_s/d_, IVS_s/d_ and LVmass, but had a 4% higher MCF (95% CI 1–7%, *p* = 0.016) which persisted after adjustment for age, sex and SEP (95% CI 1–7%, *p* = 0.008). This was attenuated to 3% after adjustment for CVD, diabetes or hypertension (all 95% CI 0–6%, all *p* < 0.070, Table 2, Fig. 1). However, no significant dose response for the number of *APOE ε*4 alleles was found in relationship with LV MCF (Table 3, Supplementary Table S3). Moreover, in NSHD + SABRE meta-analysis, *APOE ε*4 carriers had a 6% higher SV.

**Table 2:** Associations between *APOE ε4* genotypes and echocardiographic data in older age by comparing non-*APOE ε4* (*ε2ε2, ε2ε3, ε2ε3*) with *APOE ε4* (*ε2ε4*, *ε3ε4 and ε4ε4*) genotypes in the meta-analysis pooling SABRE and NSHD data

s		Model 1 (unadjusted)			Model 2 (adjusted for age, sex, and SEP)		Model 3 (Model 2 + BMI)		Model 4 (Model 2 + CVD)		Model 5 ( Model 2 + diabetes)		Model 6 (Model 2 + high cholesterol)		Model 7 (Model 2 + HT)	
**Outcome**	**APOE** ***ε4*** **status**	**n**	**Exp** ***β*** **(95% CI)**	***p*-value**	**Exp** ***β*** **(95% CI)**	***p*-value**	**Exp** ***β*** **(95% CI)**	***p*-value**	**Exp** ***β*** **(95% CI)**	***p*-value**	**Exp** ***β*** **(95% CI)**	***p*-value**	**Exp** ***β*** **(95% CI)**	***p*-value**	**Exp** ***β*** **(95% CI)**	***p*-value**
**EF**	No *APOE ε4*	ref	ref	ref	ref	ref	ref	ref	ref	ref	ref	ref	ref	ref	ref	ref
	*APOE ε4* carriers	2463	1.00 (0.98, 1.03)	0.670	1.00 (0.99, 1.02)	0.687	1.00 (0.98, 1.03)	0.677	1.00 (0.99, 1.02)	0.773	1.00 (0.98, 1.02)	0.981	1.01 (0.98, 1.03)	0.689	1.00 (0.98, 1.02)	0.697
**E/e’**	No *APOE ε4*	ref	ref	ref	ref	ref	ref	ref	ref	ref	ref	ref	ref	ref	ref	ref
	*APOE ε4* carriers	2490	0.99 (0.96, 1.01)	0.263	0.99 (0.97, 1.02)	0.453	0.99 (0.96, 1.01)	0.274	0.98 (0.96, 1.01)	0.214	0.99 (0.95, 1.03)	0.529	0.98 (0.95, 1.01)	0.116	0.99 (0.96, 1.04)	0.302
**LVmass**	No *APOE ε4*	ref	ref	ref	ref	ref	ref	ref	ref	ref	ref	ref	ref	ref	ref	ref
	*APOE ε4* carriers	2230	1.02 (0.98, 1.07)	0.347	1.01 (0.98, 1.04)	0.607	1.03 (0.98, 1.07)	0.258	1.03 (0.97, 1.09)	0.320	1.03 (0.98, 1.09)	0.249	1.02 (0.98, 1.06)	0.294	1.03 (0.98, 1.08)	0.308
**MCF**	No *APOE ε4*	ref	ref	ref	ref	ref	ref	ref	ref	ref	ref	ref	ref	ref	ref	ref
	*APOE ε4* carriers	2074	1.04 (1.01, 1.07)	**0.016**	1.04 (1.01, 1.07)	**0.008**	1.04 (1.01, 1.07)	**0.007**	1.03 (1.00, 1.06)	**0.046**	1.03 (1.00, 1.07)	0.069	1.05 (1.00, 1.09)	**0.038**	1.03 (1.00, 1.06)	**0.030**
**LVPWT**_**s**_	No *APOE ε4*	ref	ref	ref	ref	ref	ref	ref	ref	ref	ref	ref	ref	ref	ref	ref
	*APOE ε4* carriers	2476	1.00 (0.97, 1.03)	0.859	0.99 (0.97,1.02)	0.621	1.00 (0.97, 1.03)	0.847	1.00 (0.96, 1.03)	0.907	1.00 (0.97, 1.04)	0.891	1.00 (0.97, 1.03)	0.971	1.00 (0.97, 1.03)	0.882
**LVPWT**_**d**_	No *APOE ε4*	ref	ref	ref	ref	ref	ref	ref	ref	ref	ref	ref	ref	ref	ref	ref
	*APOE ε4* carriers	2488	1.00 (0.97, 1.04)	0.780	1.00 (0.98, 1.02)	0.967	1.01 (0.97, 1.04)	0.745	1.00 (0.97, 1.04)	0.805	1.01 (0.98, 1.04)	0.710	1.00 (0.98, 1.02)	0.807	1.01 (0.97, 1.04)	0.751
**IVS**_**s**_	No *APOE ε4*	ref	ref	ref	ref	ref	ref	ref	ref	ref	ref	ref	ref	ref	ref	ref
	*APOE ε4* carriers	2478	1.00 (0.98, 1.01)	0.843	0.99 (0.98, 1.01)	0.480	0.99 (0.98, 1.01)	0.478	1.00 (0.99, 1.02)	0.262	1.00 (0.99, 1.02)	0.881	0.99 (0.98, 1.01)	0.518	1.00 (0.99, 1.02)	0.992
**IVS**_**d**_	No *APOE ε4*	ref	ref	ref	ref	ref	ref	ref	ref	ref	ref	ref	ref	ref	ref	ref
	*APOE ε4* carriers	2490	1.00 (0.98, 1.02)	0.776	1.00 (0.98, 1.02)	0.972	1.00 (0.99, 1.02)	0.758	1.00 (0.99, 1.02)	0.644	1.01 (0.99, 1.03)	0.389	1.00 (0.98, 1.03)	0.759	1.00 (0.99, 1.02)	0.605

All reported analyses here consisted of random-effects meta-analyses of coefficients derived from generalized linear models with gamma distribution and log link from both NSHD and SABRE. Significant *p*-values are highlighted in bold

*β *Beta regression coefficient, *CI *Confidence interval, *exp *Exponentiated, *ref *Reference. Other abbreviations as in Table 1

**Table 3:** Dose response of *APOE ε4* carriage when assessing the association between *APOE ε4* genotype and echocardiographic and CMR data in older age

			Model 1 (unadjusted)			Model 2 (adjusted for age, sex and SEP)		Model 3 (Model 2 + BMI)		Model 4 (Model 2 + CVD)		Model 5 (Model 2 + diabetes)		Model 6 (Model 2 + high cholesterol)		Model 7 (Model 2 + HT)	
**Outcome:**	**Cohort**	**Analysis**	**n**	**Exp** ***β*** **(95% CI)**	***p*-value**	**Exp** ***β*** **(95% CI)**	***p*-value**	**Exp** ***β*** **(95% CI)**	***p*-value**	**Exp** ***β*** **(95% CI)**	***p*-value**	**Exp** ***β*** **(95% CI)**	***p*-value**	**Exp** ***β*** **(95% CI)**	***p*-value**	**Exp** ***β*** **(95% CI)**	***p*-value**
**MCF**	**UK biobank**	*APOE ε4-linear*	32,644	1.01 (1.00, 1.02)	0.082	1.01 (1.00, 1.02)	0.152	1.01 (1.00, 1.02)	0.239	1.01 (1.00, 1.02)	**0.036**	1.01 (1.00, 1.02)	0.123	1.01 (1.00, 1.02)	**0.006**	1.01 (1.00, 1.02)	0.109
	**SABRE + NSHD meta-analysis**	*APOE ε4-linear*	2074	1.02 (0.96, 1.08)	0.544	1.02 (0.96, 1.08)	0.516	1.03 (0.97, 1.09)	0.906	1.01 (0.95, 1.07)	0.729	1.00 (0.95, 1.07)	0.870	1.01 (0.94, 1.08)	0.780	1.01 (0.96, 1.07)	0.670
	**ALSPAC**	*APOE ε4-linear*	1325	1.00 (0.97, 1.03)	0.984	1.02 (0.96, 1.08)	0.604	1.01 (0.95, 1.07)	0.74	1.00 (0.94, 1.06)	0984	1.00 (0.95, 1.06)	0.310	0.99 (0.93, 1.05)	0.685	1.01 (0.94, 1.08)	0.874
	**UK biobank**	*APOE ε4* -quadratic	32,644	1.00 (0.99, 1.01)	0.770	1.00 (1.00, 1.01)	0.424	1.00 (0.99, 1.01)	0.972	1.00 (1.00, 1.01)	0.687	1.00 (1.00, 1.01)	0.711	1.00 (1.00, 1.01)	0.723	1.00 (0.99, 1.01)	0.803
	**SABRE + NSHD meta-analysis**	*APOE ε4* -quadratic	2074	0.98 (0.93, 1.03)	0.475	0.98 (0.93, 1.03)	0.451	0.99 (0.94, 1.04)	0.675	0.98 (0.94, 1.02)	0.327	0.98 (0.94, 1.02)	0.251	0.97 (0.92, 1.01)	0.174	0.98 (0.94, 1.02)	0.312
	**ALSPAC**	*APOE ε4* -quadratic	1325	0.99 (0.98, 1.00)	0.286	1.00 (0.96, 1.04)	0.865	0.98 (0.95, 1.02)	0.281	098 (0.95, 1.02)	0.286	0.98 (0.95, 1.02)	0.326	0.97 (0.94, 1.01)	0.149	0.99 (0.95, 1.03)	0.537
**Longitudinal PDSR**	**UK biobank**	*APOE ε4-linear*	32,505	1.01 (0.98, 1.04)	0.557	1.00 (0.97, 1.03)	0.855	1.01 (0.97, 1.04)	0.756	1.01 (0.98, 1.04)	0.476	1.01 (0.98, 1.04)	0.678	1.02 (0.99, 1.05)	0.199	1.01 (0.97, 1.04)	0.630
	**UK biobank**	*APOE ε4* -quadratic		0.99 (0.97, 1.01)	0.499	1.00 (0.98, 1.02)	0.640	0.99 (0.97, 1.01)	0.388	0.99 (0.97, 1.02)	0.524	0.99 (0.97, 1.02)	0.524	0.99 (0.97, 1.02)	0.526	0.99 (0.97, 1.01)	0.477
**Radial PDSR**	**UK biobank**	*APOE ε4-linear*	32,505	0.98 (0.88, 1.09)	0.725	1.02 (0.92, 1.13)	0.786	0.99 (0.89, 1.10)	0.282	0.97 (0.87, 1.08)	0.614	0.99 (0.89, 1.10)	0.858	0.95 (0.85, 1.06)	0.321	0.99 (0.88, 1.10)	0.789
	**UK biobank**	*APOE ε4* -quadratic		1.03 (0.96, 1.11)	0.379	1.02 (0.96, 1.10)	0.503	1.03 (0.96, 1.11)	0.416	1.03 (0.96, 1.11)	0.405	1.03 (0.96, 1.11)	0.400	1.03 (0.96, 1.11)	0.399	1.04 (0.96, 1.11)	0.365

The APOE *ε4* genotypes were coded as an ordered category based on the number of *ε4* possessed. Thus, level 0 encompassed *ε2ε2, ε2ε3, ε2ε3*, level 1 *ε2ε4* and *ε3 ε4* and level 2 *ε4ε4*. Given the existence of two levels, generalized linear models and orthogonal polynomial contrasts with 2 equally spaced levels (i.e., linear and quadratic) were employed to look for a dose response by *ε4* variants. Significant *p*-values are highlighted in bold. Abbreviations as in Table 2

In ALSPAC, *APOE ε*4 carriers had a 2% higher MCF (95% CI 0–5%) albeit it was not statistically significant *p* = 0.059 (Table 4). In addition, *ε*4 carriers had a 2% lower IVSd (*p* = 0.057) and LVPWT_d_ (*p* = 0.064), although these results were also not significant.

**Table 4:** Associations between *APOE ε4* genotypes and echocardiographic data in older age by comparing non-*APOE ε4* (*ε2ε2, ε2ε3, ε2ε3*) with *APOE ε4* (*ε2ε4*, *ε3ε4 and ε4ε4*) genotypes in ALSPAC

s		Model 1 (unadjusted)			Model 2 (adjusted for age, sex, and SEP)		Model 3 (Model 2 + BMI)		Model 4 (Model 2 + CVD)		Model 5 (Model 2 + diabetes)		Model 6 (Model 2 + high cholesterol)		Model 7 (Model 2 + HT)	
**Outcome**	**APOE** ***ε4*** **status**	**n**	**Exp** ***β*** **(95% CI)**	***p*-value**	**Exp** ***β*** **(95% CI)**	***p*-value**	**Exp** ***β*** **(95% CI)**	***p*-value**	**Exp** ***β*** **(95% CI)**	***p*-value**	**Exp** ***β*** **(95% CI)**	***p*-value**	**Exp** ***β*** **(95% CI)**	***p*-value**	**Exp** ***β*** **(95% CI)**	***p*-value**
**EF**	No *APOE ε4*	ref	ref	ref	ref	ref	ref	ref	ref	ref	ref	ref	ref	ref	ref	ref
	*APOE ε4* carriers	1327	1.00 (0.99, 1.01)	0.905	1.00 (0.99, 1.01)	0.737	1.00 (0.99, 1.01)	0.891	1.00 (0.99, 1.01)	0.953	1.00 (0.99, 1.01)	0.899	1.00 (0.99, 1.01)	0.890	1.00 (0.98, 1,01)	0.745
**E/e’**	No *APOE ε4*	ref	ref	ref	ref	ref	ref	ref	ref	ref	ref	ref	ref	ref	ref	ref
	*APOE ε4* carriers	1333	1.01 (0.98, 1.03)	0.531	1.01 (0.98, 1.03)	0.641	1.01 (0.98, 1.03)	0.540	1.01 (0.98, 1.03)	0.566	1.01 (0.98, 1.03)	0.578	1.01 (0.98, 1.03)	0.619	1.01 (0.99, 1.04)	0.354
**LVmass**	No *APOE ε4*	ref	ref	ref	ref	ref	ref	ref	ref	ref	ref	ref	ref	ref	ref	ref
	*APOE ε4* carriers	1333	0.98 (0.95, 1.01)	0.171	1.01 (0.98, 1.04)	0.501	0.97 (0.94, 1.00)	0.067	0.98 (0.95, 1.01)	0.171	0.98 (0.95, 1.01)	0.173	0.98 (0.94, 1.01)	0.172	0.98 (0.95, 1.02)	0.248
**MCF**	No *APOE ε4*	ref	ref	ref	ref	ref	ref	ref	ref	ref	ref	ref	ref	ref	ref	ref
	*APOE ε4* carriers	1325	1.02 (1.00, 1.05)	0.059	1.01 (0.99, 1.04)	0.270	1.03 (1.00, 1.05)	**0.036**	1.02 (1.00, 1.05)	0.060	1.02 (0.99, 1.05)	0.064	1.03 (1.00, 1.06)	0.057	1.02 (0.99, 1.05)	0.169
**LVPWT**_**s**_	No *APOE ε4*	ref	ref	ref	ref	ref	ref	ref	ref	ref	ref	ref	ref	ref	ref	ref
	*APOE ε4* carriers	1335	0.99 (0.98, 1.01)	0.406	1.00 (0.99, 1.02)	0.784	0.99 (0.98, 1.01)	0.268	0.99 (0.98, 1.01)	0.364	0.99 (0.98, 1.01)	0.413	1.00 (0.98, 1.01)	0.627	0.99 (0.98, 1.01)	0.404
**LVPWT**_**d**_	No *APOE ε4*	ref	ref	ref	ref	ref	ref	ref	ref	ref	ref	ref	ref	ref	ref	ref
	*APOE ε4* carriers	1335	0.98 (0.97, 1.00)	0.064	1.00 (0.98, 1.01)	0.631	0.98 (0.97, 1.00)	**0.032**	0.98 (0.97, 1.00)	0.059	0.98 (0.97, 1.00)	0.062	0.98 (0.96, 1.00)	**0.029**	0.99 (0.97, 1.01)	0.211
**IVS**_**s**_	No *APOE ε4*	ref	ref	ref	ref	ref	ref	ref	ref	ref	ref	ref	ref	ref	ref	ref
	*APOE ε4* carriers	1340	0.99 (0.98, 1.01)	0.415	1.00 (0.99, 1.02)	0.743	0.99 (0.97, 1.00)	0.242	0.99 (0.98, 1.01)	0.419	0.99 (0.98, 1.01)	0.422	0.99 (0.98, 1.01)	0.469	0.99 (0.97, 1.01)	0.409
**IVS**_**d**_	No *APOE ε4*	ref	ref	ref	ref	ref	ref	ref	ref	ref	ref	ref	ref	ref	ref	ref
	*APOE ε4* carriers	1340	0.98 (0.97, 1.00)	0.057	1.00 (0.98, 1.01)	0.605	0.98 (0.96, 1.00)	**0.0231**	0.98 (0.97, 1.00)	0.063	0.98 (0.97, 1.00)	0.063	0.99 (0.97, 1.00)	0.125	0.98 (0.96, 1.00)	0.085

All reported analyses here consisted of random-effects meta-analyses of coefficients derived from generalized linear models with gamma distribution and log link from both NSHD and SABRE. Significant *p*-values are highlighted in bold. Abbreviations as in Tables 1 and 2

In the sensitivity analysis, only heterozygous-*APOE ε*4 carriers had a 4% higher MCF (95% CI 1–7%, *p* = 0.016) which persisted after adjusting for sex and SEP (95% CI 1–7%, *p* = 0.013), and BMI (95% CI 1–7%, *p* = 0.018), but was attenuated to 3% after adjusting for CVD (95% CI 0–6%, *p* = 0.043, diabetes (95 CI % 0–7%, *p* = 0.060), or hypertension (95% CI 0–6%, *p* = 0.028, Table 5, Supplementary Table S4) in the meta-analysis. Similarly, in ALSPAC only heterozygous *ε*4 carriers had a higher MCF when compared to non-carriers.

**Table 5:** Associations between *APOE ε4* genotypes and echocardiographic data in older age by comparing non-*APOE ε4* (*ε2ε2, ε2ε3, ε2ε3*) with heterozygous-*APOE ε4* (*ε2ε4* and *ε3ε4*) and homozygous-*APOE ε4* (*ε4ε4*) genotypes

			Model 1 (unadjusted)			Model 2 (adjusted for sex and SEP)		Model 3 (Model 2 + BMI)		Model 4 (Model 2 + CVD)		Model 5 (Model 2 + diabetes)		Model 6 (Model 2 + high cholesterol)		Model 7 (Model 2 + HT)	
**Outcome:**	**Cohort**	**Analysis**	**n**	**Exp** ***β*** **(95% CI)**	***p*-value**	**Exp** ***β*** **(95% CI)**	***p*-value**	**Exp** ***β*** **(95% CI)**	***p*-value**	**Exp** ***β*** **(95% CI)**	***p*-value**	**Exp** ***β*** **(95% CI)**	***p*-value**	**Exp** ***β*** **(95% CI)**	***p*-value**	**Exp** ***β*** **(95% CI)**	***p*-value**
**MCF**	**UK Biobank**	Heterozygous-*APOE ε4*	31,909	1.01 (1.00, 1.01)	**0.047**	1.00 (0.99, 1.01)	0.140	1.00 (1.00, 1.01)	0.120	1.01 (1.00, 1.01)	**0.019**	1.00 (1.00, 1.01)	0.116	1.01 (1.00, 1.01)	**0.0008**	1.00 (1.00, 1.01)	0.069
	**SABRE + NSHD meta-analysis**	Heterozygous-*APOE ε4*	2019	1.04 (1.01, 1.07)	**0.016**	1.04 (1.01, 1.07)	**0.013**	1.04 (1.01, 1.07)	**0.018**	1.03 (1.00, 1.06)	**0.043**	1.03 (1.00, 1.07)	0.060	1.05 (1.00, 1.10)	**0.040**	1.03 (1.00, 1.06)	**0.028**
	**ALSPAC**	Heterozygous-*APOE ε4*	1328	1.02 (1.00, 1.05)	0.066	1.02 (0.99, 1.04)	0.236	1,03 (1.00, 1.05)	**0.039**	1.02 (1.00, 1.05)	0.067	1.02 (0100, 1.05)	0.069	1.03 (1.00, 1.06)	0.079	1.02 (0.99, 1.05)	0.169
	**UK Biobank**	Homozygous-*APOE ε4*	25,086	1.01 (1.00, 1.03)	0.083	1.01 (1.00, 1.02)	0.166	1.01 (1.00, 1.02)	0.252	1.01 (1.00, 1.03)	**0.034**	1.01 (1.00, 1.02)	0.115	1.02 (1.01, 1.03)	**0.006**	1.01 (1.00, 1.02)	0.123
	**SABRE + NSHD meta-analysis**	Homozygous-*APOE ε4*	1539	1.03 (0.95, 1.11)	0.544	1.03 (0.95, 1.11)	**0.517**	1.04 (0.96, 1.13)	0.350	1.02 (0.92, 1.10)	0.704	1.01 (0.93, 1.09)	0.874	1.01 (0.92, 1.11)	0.812	1.02 (0.94, 1.10)	0.652
	**ALSPAC**	Homozygous-*APOE ε4*	998	1.00 (0.96, 1.05)	0.984	1.01 (0.97, 1.06)	0.588	1.00 (0.96, 1.05)	0.892	1.00 (0.96, 1.05)	0.984	1.00 (0.96, 1.05)	0.935	0.99 (0.95, 1.04)	0.701	1.00 (0.95, 1.06)	0.920
**Longitudinal PDSR**	**UK biobank**	Heterozygous-*APOE ε4*	31,909	1.02 (1.00, 1.03)	**0.049**	1.00 (0.99, 1.02)	0.610	1.02 (1.00, 1.03)	0.059	1.02 (1.00, 1.03)	**0.038**	1.01 (1.00, 1.03)	0.099	1.02 (1.01, 1.04)	**0.004**	1.02 (1.00, 1.03)	0.062
	**UK biobank**	Heterozygous-*APOE ε4*	24,965	1.01 (0.97, 1.06)	0.556	1.00 (0.96, 1.04)	0.843	1.01 (0.96, 1.05)	0.754	1.02 (0.97, 1.06)	0.469	1.01 (0.97, 1.06)	0.679	1.03 (0.98, 1.08)	0.206	1.01 (0.97, 1.06)	0.631
**Radial PDSR**	**UK biobank**	Heterozygous-*APOE ε4*	31,773	0.95 (0.90, 1.00)	**0.049**	0.98 (0.93, 1.03)	0.467	0.96 (0.90, 1.01)	0.094	0.94 (0.89, 1.00)	**0.035**	0.96 (0.91, 1.01)	0.097	0.93 (0.88, 0.98)	**0.005**	0.95 (0.90, 1.00)	0.058
	**UK biobank**	Heterozygous-*APOE ε4*	24,965	0.97 (0.83, 1.14)	0.726	1.02 (0.88, 1.18)	0.772	0.98 (0.84, 1.15)	0.820	0.96 (0.82, 1.12)	0.605	0.99 (0.85, 1.15)	0.859	0.93 (0.80, 1.08)	0.327	0.98 (0.84, 1.14)	0.789

All reported analyses here consisted of generalized linear models with gamma distribution and log link. Significant *p*-values are highlighted in bold. Abbreviations as in Table 2

In ALSPAC, NSHD, or SABRE, neither a linear nor a quadratic dose effect based on the number of *ε*4 alleles was observed. The association between *ε*4 and MCF in the SABRE + NSHD meta-analysis persisted at an FDR of 0.15.

### Associations between *APOE ε4* genotypes and CMR data

In UK Biobank, when compared to the non-*APOE ε*4 group, *APOE ε*4 carriers had a 1% higher MCF 95% (CI 0–1%, *p* = 0.020) which persisted after adjusting for age, sex and SEP (Model 2, *p* = 0.080), CVD (Model 4, *p* = 0.006), high cholesterol (Model 5, *p* = 0.0001) or hypertension (Model 7, *p* = 0.034), but was attenuated to 0% (95% CI 0–1%) after adjusting for BMI (Model 3, *p* = 0.079) or diabetes (*p* = 0.058, Table 6, Fig. 1). There was a dose-response relationship based on the number of *ε*4 alleles, especially when adjusting for CVD in Model 4 (*p* = 0.036) and high cholesterol in Model 6 (*p* = 0.006, Table 3). However, although heterozygous-*APOE ε*4 carriers had a higher MCF, the association was not significant for homozygous-*APOE ε*4 carriers (Table 5).

**Table 6:** Associations between *APOE ε4* genotypes and echocardiographic data in older age by comparing non-*APOE ε4* (*ε2ε2, ε2ε3, ε2ε3*) with *APOE ε4* (*ε2ε4*, *ε3ε4 and ε4ε4*) genotypes in UK Biobank

s		Model 1 (unadjusted)			Model 2 (adjusted for age, sex, and SEP)		Model 3 (Model 2 + BMI)		Model 4 (Model 2 + CVD)		Model 5 (Model 2 + diabetes)		Model 6 (Model 2 + high cholesterol)		Model 7 (Model 2 + HT)	
**Outcome**	**APOE** ***ε4*** **status**	**n**	**Exp** ***β*** **(95% CI)**	***p*-value**	**Exp** ***β*** **(95% CI)**	***p*-value**	**Exp** ***β*** **(95% CI)**	***p*-value**	**Exp** ***β*** **(95% CI)**	***p*-value**	**Exp** ***β*** **(95% CI)**	***p*-value**	**Exp** ***β*** **(95% CI)**	***p*-value**	**Exp** ***β*** **(95% CI)**	***p*-value**
**EF**	No *APOE ε4*	ref	ref	ref	ref	ref	ref	ref	ref	ref	ref	ref	ref	ref	ref	ref
	*APOE ε4* carriers	32,644	1.00 (1.00, 1.00)	0.916	1.00 (1.00, 1.00)	0.453	1.00 (1.00, 1.00)	0.902	1.00 (1.00, 1.00)	0.836	1.00 (1.00, 1.00)	0.830	1.00 (1.00, 1.00)	0.840	1.00 (1.00, 1.00)	0.914
**L**_**PDSR**_	No *APOE ε4*	ref	ref	ref	ref	ref	ref	ref	ref	ref	ref	ref	ref	ref	ref	ref
	*APOE ε4* carriers	32,505	1.02 (1.00, 1.03)	**0.045**	1.00 (0.99, 1.02)	0.657	1.02 (1.00, 1.03)	0.063	1.02 (1.00, 1.03)	**0.032**	1.01 (1.00, 1.03)	0.095	1.02 (1.01, 1.04)	**0.002**	1.02 (1.00, 1.03)	0.059
**R**_**PDSR**_	No *APOE ε4*	ref	ref	ref	ref	ref	ref	ref	ref	ref	ref	ref	ref	ref	ref	ref
	*APOE ε4* carriers	32,505	0.95 (0.90, 1.00)	**0.05**	0.98 (0.94, 1.04)	0.536	0.96 (0.91, 1.01)	0.102	0.95 (0.90, 1.00)	**0.034**	0.96 (0.91, 1.01)	0.106	0.93 (0.88, 0.98)	**0.004**	0.95 (0.90, 1.00)	0.063
**LVmass**	No *APOE ε4*	ref	ref	ref	ref	ref	ref	ref	ref	ref	ref	ref	ref	ref	ref	ref
	*APOE ε4* carriers	32,644	1.00 (0.99, 1.01)	0.568	1.00 (1.00, 1.01)	0.242	1.00 (0.99, 1.01)	0.798	1.00 (0.99, 1.00)	0.350	1.00 (0.99, 1.01)	0.815	1.00 (0.99, 1.00)	0.127	1.00 (0.99, 1.01)	0.695
**MCF**	No *APOE ε4*	ref	ref	ref	ref	ref	ref	ref	ref	ref	ref	ref	ref	ref	ref	ref
	*APOE ε4* carriers	32,643	1.01 (1.00, 1.01)	**0.020**	1.01 (1.00, 1.01)	0.080	1.00 (1.00, 1.01)	0.079	1.01 (1.00, 1.01)	**0.006**	1.00 (1.00, 1.01)	0.058	1.01 (1.01, 1.01)	**0.0001**	1.01 (1.00, 1.01)	**0.034**
**PWT**	No *APOE ε4*	ref	ref	ref	ref	ref	ref	ref	ref	ref	ref	ref	ref	ref	ref	ref
	*APOE ε4* carriers	32,605	1.00 (0.99, 1.00)	0.094	1.00 (1.00, 1.00)	0.881	1.00 (1.00, 1.00)	0.448	1.00 (0.99, 1.00)	**0.037**	1.00 (1.00, 1.00)	0.257	0.99 (0.99, 1.00)	**0.002**	1.00 (1.00, 1.00)	0.132
**IVS**	No *APOE ε4*	ref	ref	ref	ref	ref	ref	ref	ref	ref	ref	ref	ref	ref	ref	ref
	*APOE ε4* carriers	32,605	1.00 (0.99, 1.00)	0.128	1.00 (1.00, 1.00)	0.986	1.00 (1.00, 1.00)	0.537	1.00 (0.99, 1.00)	0.056	1.00 (0.99, 1.00)	0.281	1.00 (0.99, 1.00)	**0.003**	1.00 (0.99, 1.00)	0.179

All reported analyses here consisted of generalized linear models with gamma distribution and log link, except for the longitudinal and radial PDSR analyses where generalized linear models with Gaussian distribution and identity link were used instead. Significant *p*-values are highlighted in bold. Abbreviations as in Tables 1 and 2

In addition, *APOE ε*4 carriers had a 2% higher longitudinal PDSR (95% CI 0–3%, *p* = 0.045), which persisted after adjusting for CVD and diabetes, but was attenuated to 0% in Model 2 and to 1% after adjusting for diabetes (Model 5). Conversely, they had a 5% lower radial PDSR (95% CI 0.90–1.00, *p* = 0.05) which behaved similar to longitudinal PDSR on adjustment (Table 6).

The associations between *ε*4 carriage and MCF, radial and longitudinal PDSR persisted at an FDR of 0.15 in the UK Biobank.

## Discussion

**Fig. 2:**
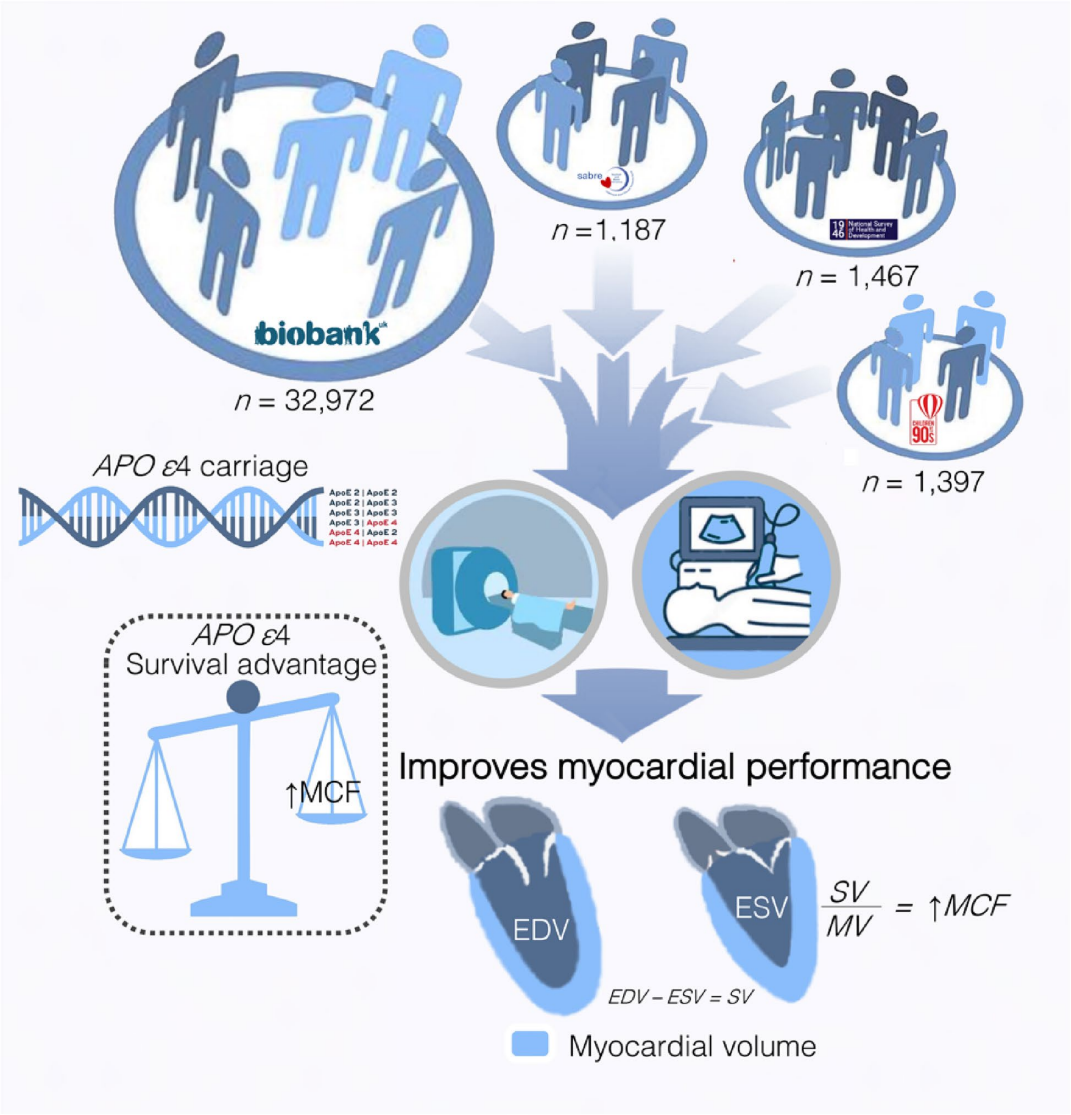
Graphical abstract. Combining data from four British cohorts–1946 National Survey of Health and Development (NSHD), Southall and Brent Revised (SABRE), UK Biobank and Avalon Longitudinal Study of Parents and Children (ALSPAC)–we explored whether *APOE ε*4 carriage associates with beneficial or unfavorable left ventricular (LV) structural and functional parameters by echocardiography and cardiovascular magnetic resonance (CMR). Based on the presence of *APOE ε*4, genotypes were divided into: *APOE ε*4 (ε2ε4, ε3ε4, *ε*4*ε*4) and non-*APOE ε*4 carriers. Compared to the non-*APOE ε*4 group, *APOE ε*4 carriers had a higher myocardial contraction fraction resulting in greater LV stroke volume generation per 1 mL of myocardium and better longitudinal strain rates compared to non *APOE ε*4 carriers

Data from 37,000 young and older British persons show that *APOE ε*4 carriage associates with slightly advantageous myocardial performance manifesting as higher MCF and longitudinal strain rates, but slightly lower radial strain rates. A graphical abstract of this work is presented in Fig. 2.

*APOE ε*4 might be another example of antagonistic pleiotropy [6] as *ε*4 carriage appears to be both beneficial (e.g., fertility and resistance to infections [7]) and detrimental (e.g., Alzheimer’s disease) to human health. The occurrence of the latter further down the fertility timeline in older age might explain the allele’s persistence in spite of natural selection.

In terms of cardiovascular health, *APOE ε*4 carriage was previously associated with CVD (IHD [14] and myocardial infarction [36]) and CVD risk factors (such as hypertension [12] and diabetes [13]). Although the exact mechanism is yet to be elucidated, it is postulated that *APOE ε*4 might contribute to the development of metabolic syndrome [37]. *APOE ε*4 differs from *APOE ε*3 at amino acid position 112 where arginine (positively charged side chain) is present instead of cysteine (non-polar side chain). Given its ability to bind to peripheral and hepatic lipoprotein receptors, it is plausible for the *APOE ε* isoforms to have different binding affinities explaining the link with dyslipidemia [14]. However, emerging evidence points to more a complex mechanism as *APOE ε* can also alter the levels of *APOB* [38] which is itself also associated with CVD [39]. In addition, *APOE ε* is mainly produced by the liver, but can also be synthesized in and regulate the activity of adipocytes [40] which might explain the relationship between *APOE ε4* and insulin resistance [37, 41].

Here we show that *APOE ε*4 carriage appears to associate with a higher MCF. The MCF is a volumetric index of LV myocardial shortening which captures maladaptive myocardial hypertrophy otherwise missed by conventional biomarkers such as EF, mass, and wall thickness, as it considers the relationship between LVmass and SV [42]. It has been previously associated with CV morbidity and mortality independent of conventional risk factors [43]. In addition, it is regarded as a highly-sensitive metric of systolic function, and low values have been linked to negative outcomes even in the presence of apparently normal LV EF [44] indicating its strength as a subclinical disease marker. Interestingly, MCF was higher in CMR compared to echocardiography since the later underestimates LV volumes such as stroke volume [45]. A higher MCF in the context of *APOE ε*4 carriage might mean a slightly advantageous cardiac phenotype in terms of heart function. Dissociable effects of *APOE ε*4 carriage have been previously reported in the context of better attention despite the higher risk of Alzheimer’s disease [10]. Although the literature is sparse, *APOE ε*4 carriage has been previously linked to higher levels of androgens [46] or dysregulated glucose and ketone metabolism [7] which could putatively increase myocardial contractility leading to a higher stroke volume per unit of LV mass which is being captured by the MCF [47].

Importantly, we found a dose response relationship for MCF based on the number of *ε*4 alleles carried by an individual in the UK Biobank (*n* = 32,972) using CMR data. This finding aligns with biological plausibility suggesting that there is a consistent relationship between *ε*4 and higher MCF. However, this dose effect relationship was not apparent in ALSPAC, NSHD or SABRE which is likely because these studies were underpowered. Indeed, the number of homozygous *ε*4 carriers were *n* = 27 for ALSPAC, *n* = 46 for NSHD and *n* = 29 for SABRE compared to *n* = 741 in the UK Biobank. Another explanation is that healthier *APOE ε*4 carriers may have been more likely to survive and/or to participate in the older age cohort studies resulting in selection bias. This would fit with the known effects of *APOE ε*4 carriage on IHD, HT, lipids, and cognitive function. Previous studies have described cognitive advantages in heterozygotes that were not replicated in the homozygotes [48] potentially mirroring some of our data.

**Fig. 3:**
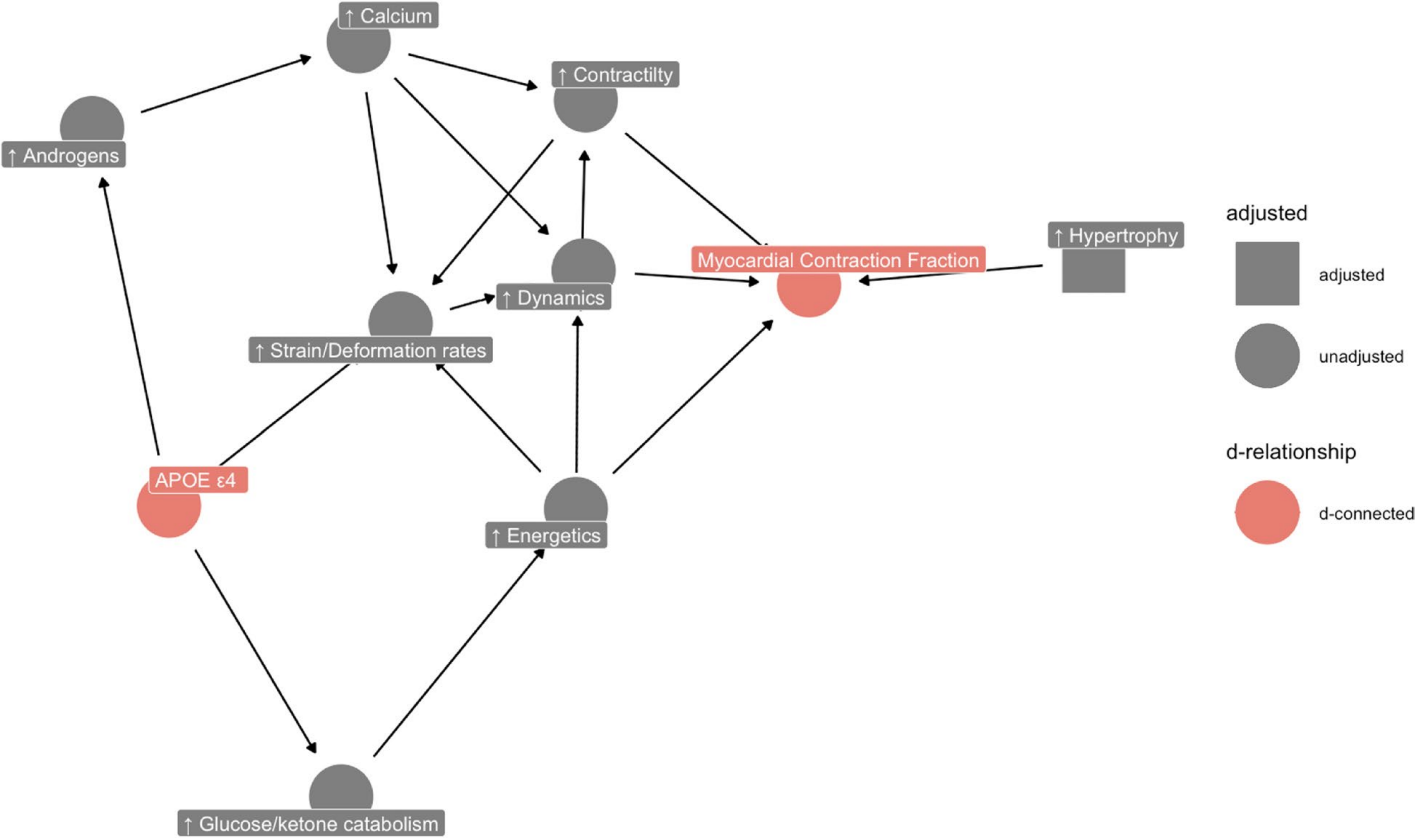
Directed acyclic graph highlighting potential mechanism underpinning the association between *APOE ε*4 and MCF. *APOE ε*4 carriers had a better strain profile characterized by higher absolute (i.e., better) longitudinal and radial PDSRs using CMR in the UK Biobank. In addition, *ε*4 carriers had a slightly lower E/e’ (in ALSPAC and NSHD) and LVmass (in SABRE and UK Biobank) albeit not statistically significant. In the literature, *ε*4 has been linked to a higher level of androgens which can increase myocardial calcium and *ε*4 has also been linked to pro-catabolic glucose and ketone metabolism. Thus, we postulate that enhanced myocardial dynamics, contractility, and energetics rather than pathological hypertrophy mediate this association

Indeed, *APOE ε*4 carriage was associated with a greater longitudinal and lower radial strain both of which are markers of a positive cardiac phenotype. This suggests that different myocardial contraction dynamics might be contributing to the observed association with MCF (Fig. 3). The observed trend linking *APOE ε*4 carriage with slightly better echocardiographic LV filling pressures (lower E/e’ may suggest less ventricular stiffness in some but not all cases [49]), albeit attenuated in multivariable models, lends plausibility to this theory. The CMR analyses indicated a slight association between *APOE ε*4 carriage and thinner ventricular walls, and similarly the echocardiographic analyses found no association between *APOE ε*4 carriage and LV hypertrophy biomarkers (LVPWT_s/d_, IVS_s/d_, LVmass). Moreover, ε4 carriage had a higher SV only in SABRE and NSHD but not in ALSPAC or UK Biobank. MCF is a dimensionless metric as the SV is divided by the myocardial volume meaning that size related contributions to these metrics cancel out. Whilst a higher SV may partly drive the larger MCF, it is the SV per 1 ml of myocardium which is improved.

Interestingly, the effect sizes capturing the association *APOE ε*4 and MCF were higher after adjusting for high cholesterol. In addition to being a precursor for androgens higher level of which were observed in ε4 carriers and which can promote contractility [46], high cholesterol in order age was linked to increased longevity due to lower mortality from cancer and infection [50]. However, since a higher MCF was also observed in ASLPAC in < 24 years individuals, the benefit is not restricted to the elderly. These data collectively suggest that the observed MCF enhancement is not mediated by pathological ventricular thickening but through improved myocardial energetics and contractility, with calcium potentially implicated [46, 47]. Since the models were attenuated after adjusting for CVD, diabetes and hypertension, the benefits which stem from *ε*4 carriage are reduced as an individual starts to develop *APOE ε*4 related negative outcomes.

The effect size of the association between *APOE ε*4 carriage and MCF was < 5% across all cohorts. Indeed, genome wide association studies (GWASs) highlighted that individual gene effects on cardiac phenotypes are usually small [51–53]. Indeed, polygenic scores which are calculated as weighted sums of SNPs may provide a more meaningful estimate of an individual’s genetic liability to cardiac disease [54].

The main strength of our study is that we were able to replicate the findings in four independent cohorts encompassing 37,000 individuals, across two imaging modalities (echocardiography and CMR) suggesting that there is an advantageous phenotype in terms of MCF in *ε4* carriers. In addition, as the MRC NSHD and ALSPAC are birth cohorts, the participants were implicitly age-matched across all the analyses, exposed to similar epoch-related risk factors and had access to similar treatment facilities across the decades. Since NSHD, SABRE and UK Biobank are longitudinal cohorts in which timing of genotyping and imaging were not necessarily contemporaneous, selective follow-up may have potentially excluded homozygous or heterozygous individuals who already passed away with the worst cardiac phenotypes. However, we managed to replicate our findings in a young cohort (< 24 years) which lends credence to the notion that *ε*4 carriage associates with an improved cardiac phenotype in terms of MCF. Although most study participants were unrelated, family ties do exist and not controlling them is a limitation of this study.

## Conclusion

*APOE ε*4 carriage associates with improved myocardial performance from adolescence to older age resulting in greater LV stroke volume generation per 1 mL of myocardium and better longitudinal strain rates compared to non *APOE ε*4 carriers. This potentially favorable cardiac phenotype adds to the growing number of reported survival advantages attributed to *APOE* ε4 carriage that might collectively explain its persistence in humans.

### Supplementary Information

**Additional file 1:** Supplementary Tables S1-S5.

## Data Availability

ALSPAC data is available from http://www.bristol.ac.uk/alspac/ . NSHD data is available from: https://www.nshd.mrc.ac.uk/data, SABRE data is available from https://www.sabrestudy.org/, and UK Biobank data is available from https://www.ukbiobank.ac.uk/ . Upon publication, the final R scripts will be made publicly available on GitHub.
