# Supplementary material for: APOE ε4 carriage associates with improved myocardial performance from adolescence to older age
Source: BMC Cardiovasc Disord. 2024 Mar 21;24:172. doi: 10.1186/s12872-024-03808-z (PMC10956279; doi:10.1186/s12872-024-03808-z)
Supplement: Supplementary file 1 — Additional file 1: Supplementary Tables S1-S5. [file 12872_2024_3808_MOESM1_ESM.pdf]

SUPPLEMENTARY MATERIAL FOR:

APOE ε4 carriage associates with improved myocardial performance from adolescence to older age

Constantin-Cristian Topriceanu[1,2,3], Mit Shah[4,5], Matthew Webber[1,2], Fiona Chan[1,2], Hunain Shiwani[2,3], Marcus Richards[1], Jonathan Schott[1,6], Nishi Chaturvedi[1,2], James C Moon [2,3], Alun D. Hughes[1,2], Aroon D Hingorani[2,7,8], Declan P. O'Regan[4,5], Gabriella Captur [1,2,3,9]

Author Affiliations:

1. UCL MRC Unit for Lifelong Health and Ageing, University College London, London, UK
2. UCL Institute of Cardiovascular Science, University College London, London, UK
3. Cardiac MRI Unit, Barts Heart Centre, London, UK
4. National Heart and Lung Institute, Imperial Centre for Translational and Experimental Medicine, Imperial College London, London, UK
5. MRC London Institute of Medical Sciences, Imperial College London, London, UK
6. Dementia Research Centre, UCL Queen Square Institute of Neurology, London, UK
7. BHF Research Accelerator, University College London, London, UK
8. Health Data Research, University College London, UK
9. The Royal Free Hospital, Centre for Inherited Heart Muscle Conditions, Cardiology Department, London, UK

Corresponding author:

Gabriella Captur

Consultant Cardiologist in Inherited Heart Muscle Conditions, Senior Clinical Lecturer

Institute of Cardiovascular Science, University College London, Gower Street, London WC1E 6BT, UK

E-mail: gabriella.captur@ucl.ac.uk, Phone No: +44 2074600595

Supplementary Table S1. Associations between *APOE ε4* genotypes and echocardiographic data by comparing non-*APOE ε4* (*ε2ε2, ε2ε3, ε2ε3*) with any *APOE ε4* (*ε2ε4, ε3ε4 and ε4ε4*) genotypes in NSHD.

s		**M1** (unadjusted)			**M2** (adjusted for sex and SEP)		**M3** (M2 + BMI)		**M4** (M2 + CVD)		**M5** (M2 + diabetes)		**M6** (**M2 + high cholesterol)		**M7** (**M2 + HT)	
**Outcome**	**APOE *ε4* status**	**n**	**Exp *β*** (95% CI)	***p*-value**	**Exp *β*** **(**95% CI)	***p*-value**	**Exp *β*** (95% CI)	***p*-value**	**Exp *β*** (95% CI)	***p*-value**	**Exp *β*** (95% CI)	***p*-value**	**Exp *β*** (95% CI)	***p*-value**	**Exp *β*** (95% CI)	***p*-value**
**EF**	No *APOE ε4*	ref	ref	ref	ref	ref	ref	ref	ref	ref	ref	ref	ref	ref	ref	ref
	APOE ε4 carriers	1303	0.99 (0.98, 1.01)	0.421	1.00 (0.98, 1.01)	0.552	0.99 (0.98, 1.01)	0.414	1.00 (0.98, 1.01)	0.524	0.99 (0.98, 1.01)	0.160	0.99 (0.98, 1.01)	0.436	1.00 (0.98, 1.01)	0.524
**E/e'**	No *APOE ε4*	ref	ref	ref	ref	ref	ref	ref	ref	ref	ref	ref	ref	ref	ref	ref
	APOE ε4 carriers	1342	0.98 (0.95, 1.01)	0.193	0.99 (0.96, 1.02)	0.288	0.98 (0.95, 1.01)	0.186	0.97 (0.94, 1.01)	0.101	0.97 (0.94, 1.00)	0.080	0.97 (0.94, 1.01)	0.115	0.98 (0.95, 1.01)	0.218
**LVmass**	No *APOE ε4*	ref	ref	ref	ref	ref	ref	ref	ref	ref	ref	ref	ref	ref	ref	ref
	APOE ε4 carriers	1069	1.05 (1.00, 1.11)	0.057	1.01 (0.97, 1.06)	0.665	1.05 (1.01, 1.1)	**0.033**	1.06 (1.00, 1.13)	**0.040**	1.07 (1.01, 1.13)	**0.024**	1.03 (0.98, 1.10)	0.270	1.06 (1.00, 1.11)	**0.040**
**MCF**	No *APOE ε4*	ref	ref	ref	ref	ref	ref	ref	ref	ref	ref	ref	ref	ref	ref	ref
	APOE ε4 carriers	916	1.06 (1.00, 1.12)	**0.050**	1.06 (1.00, 1.12)	**0.038**	1.05 (1.00, 1.11)	0.064	1.05 (0.99,1.12)	0.112	1.06 (1.00, 1.12)	0.056	1.08 (1.01, 1.14)	**0.020**	1.05 (0.99, 1.11)	0.081
**LVPWT$_s$**	No *APOE ε4*	ref	ref	ref	ref	ref	ref	ref	ref	ref	ref	ref	ref	ref	ref	ref

	APOE ε4 carriers	1315	1.01 (0.99, 1.04)	0.207	1.01 (0.99, 1.03)	0.537	1.01 (0.99, 1.03)	0.226	1.02 (0.99, 1.04)	0.152	1.02 (1.00, 1.05)	0.053	1.01 (0.99, 1.04)	0.231	1.01 (0.99, 1.04)	0.190
**$LVPWT_d$**	No *APOE ε4*	ref	ref	ref	ref	ref	ref	ref	ref	ref	ref	ref	ref	ref	ref	ref
	APOE ε4 carriers	1325	1.02 (1.00, 1.05)	0.076	1.01 (0.99, 1.04)	0.199	1.02 (1.00, 1.05)	0.075	1.02 (0.99, 1.05)	0.125	1.02 (0.99, 1.05)	0.123	1.02 (0.99, 1.05)	0.339	1.02 (1.00, 1.05)	0.066
**IVS_s**	No *APOE ε4*	ref	ref	ref	ref	ref	ref	ref	ref	ref	ref	ref	ref	ref	ref	ref
	APOE ε4 carriers	1317	1.00 (0.98, 1.02)	0.952	0.99 (0.97, 1.01)	0.394	1.00 (0.98, 1.02)	0.877	1.01 (0.98, 1.03)	0.524	1.00 (0.98, 1.03)	0.842	0.99 (0.97, 1.01)	0.432	1.00 (0.98, 1.02)	0.935
**IVS_d**	No *APOE ε4*	ref	ref	ref	ref	ref	ref	ref	ref	ref	ref	ref	ref	ref	ref	ref
	APOE ε4 carriers	1327	1.01 (0.98, 1.04)	0.442	1.00 (0.98, 1.03)	0.834	1.01 (0.98, 1.04)	0.491	1.01 (0.98, 1.05)	0.356	1.02 (0.99, 1.05)	0.239	1.00 (0.97, 1.03)	0.867	1.01 (0.98, 1.04)	0.411

All reported analyses here consisted of generalized linear models with gamma distribution and log link. Significant *p*-values are highlighted in bold.
Model 2 was not adjusted for age in NSHD as all participants are age-matched.

APOE ε4 = apolipoprotein ε4; β = regression coefficient; *BMI = body mass index;* CI = confidence interval; CVD = cardiovascular disease; *EF = ejection fraction;* exp = exponentiated; HT = hypertension; $IVSs_{/d}$,= *interventricular septal thickness in systole/diastole; LVmass = left ventricular mass, $LVPWT_{s/d}$ = left ventricular posterior wall thickness in systole/diastole; M = model; MCF = myocardial contraction fraction; NSHD = National Survey of Health and Development; PDSR = peak diastolic strain rate;* ref = reference.

Supplementary Table S2. Associations between *APOE ε4* genotypes and echocardiographic data at 60-64 years by comparing non-*APOE ε4* (*ε2ε2, ε2ε3, ε2ε3*) with any *APOE ε4* (*ε2ε4, ε3ε4 and ε4ε4*) genotypes in SABRE.

s		M1 (unadjusted)			M2 (adjusted for age, sex and SEP)		M3 (M2 + BMI)		M4 (M2 + CVD)		M5 (M2 + diabetes)		M6 (M2 + high cholesterol)		M7 (M2 + HT)	
**Outcome**	**APOE *ε4* status**	**n**	**Exp *β*** (95% CI)	***p*-value**	**Exp *β*** (95% CI)	***p*-value**	**Exp *β*** (95% CI)	***p*-value**	**Exp *β*** (95% CI)	***p*-value**	**Exp *β*** (95% CI)	***p*-value**	**Exp *β*** (95% CI)	***p*-value**	**Exp *β*** (95% CI)	***p*-value**
**EF**	No *APOE ε4*	ref	ref	ref	ref	ref	ref	ref	ref	ref	ref	ref	ref	ref	ref	ref
	APOE ε4 carriers	1160	1.02 (1.00, 1.04)	0.134	1.02 (1.00, 1.04)	0.153	1.02 (1.00, 1.04)	0.144	1.01 (0.99, 1.03)	0.228	1.01 (0.99, 1.03)	0.228	1.02 (1.00, 1.05)	0.109	1.02 (1.00, 1.04)	0.148
**E/e'**	No *APOE ε4*	ref	ref	ref	ref	ref	ref	ref	ref	ref	ref	ref	ref	ref	ref	ref
	APOE ε4 carriers	1148	1.00 (0.96, 1.04)	0.921	1.00 (0.95, 1.04)	0.894	1.01 (1.00, 1.01)	0.984	1.00 (0.96, 1.04)	0.999	1.01 (0.97, 1.06)	0.651	0.99 (0.93, 1.05)	0.679	1.03 (1.01, 1.06)	0.968
**LVmass**	No *APOE ε4*	ref	ref	ref	ref	ref	ref	ref	ref	ref	ref	ref	ref	ref	ref	ref
	APOE ε4 carriers	1161	1.00 (0.97, 1.04)	0.918	1.01 (0.97, 1.04)	0.752	1.01 (0.97, 1.04)	0.710	1.00 (0.97, 1.04)	0.829	1.01 (0.97, 1.05)	0.656	1.01 (0.96, 1.06)	0.657	1.00 (0.97, 1.04)	0.846
**MCF**	No *APOE ε4*	ref	ref	ref	ref	ref	ref	ref	ref	ref	ref	ref	ref	ref	ref	ref
	APOE ε4 carriers	1158	1.03 (1.00, 1.06)	0.102	1.03 (1.00, 1.07)	0.0961	1.03 (1.00, 1.07)	0.084	1.02 (0.99, 1.06)	0.161	1.02 (0.99, 1.06)	0.243	1.03 (0.99, 1.07)	0.211	1.03 (0.99, 1.06)	0.141
**LVPWT$_s$**	No *APOE ε4*	ref	ref	ref	ref	ref	ref	ref	ref	ref	ref	ref	ref	ref	ref	ref
	APOE ε4 carriers	1161	0.98 (0.96, 1.00)	0.058	0.98 (0.96, 1.00)	0.067	0.98 (0.97, 1.00)	0.0571	0.98 (0.96, 1.00)	**0.046**	0.98 (0.97, 1.00)	0.104	0.99 (0.96, 1.01)	0.274	0.98 (0.96, 1.00)	0.065

**LVPWT$_d$**	No *APOE ε4*	ref	ref	ref	ref	ref	ref	ref	ref	ref	ref	ref	ref	ref	ref	ref
	APOE ε4 carriers	1163	0.99 (0.97, 1.01)	0.268	0.99 (0.97, 1.01)	0.308	0.99 (0.97, 1.01)	0.314	0.99 (0.97, 1.01)	0.289	0.99 (0.97, 1.01)	0.454	0.98 (0.95, 1.01)	0.581	0.99 (0.97, 1.01)	0.293
**IVS$_s$**	No *APOE ε4*	ref	ref	ref	ref	ref	ref	ref	ref	ref	ref	ref	ref	ref	ref	ref
	APOE ε4 carriers	1161	1.00 (0.98, 1.02)	0.829	1.00 (0.98, 1.02)	0.885	1.00 (0.98, 1.02)	0.934	1.00 (0.98, 1.02)	0.836	1.00 (0.98, 1.02)	0.983	1.00 (0.98, 1.03)	0.946	1.00 (0.98, 1.02)	0.948
**IVS$_d$**	No *APOE ε4*	ref	ref	ref	ref	ref	ref	ref	ref	ref	ref	ref	ref	ref	ref	ref
	APOE ε4 carriers	1163	1.00 (0.97, 1.02)	0.782	1.00 (0.97, 1.02)	0.822	1.00 (0.98, 1.02)	0.856	1.00 (0.98, 1.02)	0.899	1.00 (0.98, 1.03)	0.862	1.00 (0.97, 1.04)	0.788	1.00 (0.98, 1.02)	0.986

All reported analyses here consisted of generalized linear models with gamma distribution and log link. Significant *p*-values are highlighted in bold.

SABRE, Southall and Brent Revised. Other abbreviations as in **Supplementary Table S1**.

Supplementary Table S3. Dose response of *APOE ε4* carriage when assessing the association between *APOE ε4* genotype and echocardiographic data in NSHD.

				**M1** (unadjusted)		**M2** (adjusted for age, sex and SEP)		**M3** (M2 + BMI)		**M4** (M2 + CVD)		**M5** (M2 + diabetes)		**M6** (M2 + high cholesterol)		**M7** (M2 + HT)	
**Outcome**	**Cohort**	**Analysis**	**n**	**Exp *β*** (95% CI)	***p*-value**	**Exp *β*** (95% CI)	***p*-value**	**Exp *β*** (95% CI)	***p*-value**	**Exp *β*** (95% CI)	***p*-value**	**Exp *β*** (95% CI)	***p*-value**	**Exp *β*** (95% CI)	***p*-value**	**Exp *β*** (95% CI)	***p*-value**
**MCF**	**SABRE**	*APOE ε4-linear*	158	1.00 (0.94, 1.08)	0.901	1.01 (0.94, 1.08)	0.878	1.01 (0.94, 1.08)	0.779	1.00 (0.94, 1.07)	0.974	1.00 (0.94, 1.07)	0.984	1.01 (0.93, 1.10)	0.873	1.00 (0.94, 1.07)	0.985
	**NSHD**	*APOE ε4-linear*	916	1.05 (0.95, 1.17)	0.354	1.05 (0.95, 1.17)	0.340	1.06 (0.96, 1.18)	0.230	1.04 (0.93, 1.17)	0.525	1.02 (0.91, 1.14)	0.775	1.02 (0.91, 1.14)	0.802	1.04 (0.94, 1.16)	0.452
	**SABRE +NSHD**	Meta-analysis (linear)	2074	1.02 (0.96, 1.08)	0.544	1.02 (0.96, 1.08)	0.516	1.03 (0.97, 1.09)	0.906	1.01 (0.95, 1.07)	0.729	1.00 (0.95, 1.07)	0.870	1.01 (0.94, 1.08)	0.780	1.01 (0.96, 1.07)	0.670
	**SABRE**	*APOE ε4* - quadratic	1158	0.98 (0.93, 1.03)	0.350	0.98 (0.93, 1.03)	0.356	0.98 (0.94, 1.03)	0.404	0.98 (0.94, 1.03)	0.381	0.98 (0.94, 1.03)	0.460	0.98 (0.93, 1.04)	0.499	0.98 (0.93, 1.03)	0.350
	**NSHD**	*APOE ε4* - quadratic	916	0.99 (0.92, 1.06)	0.713	0.98 (0.92, 1.06)	0.664	1.00 (0.93, 1.07)	0.980	0.98 (0.91, 1.07)	0.660	0.96 (0.89, 1.04)	0.315	0.95 (0.88, 1.03)	0.171	0.99 (0.92, 1.06)	0.676
	**SABRE +NSHD**	Meta-analysis (quadratic)	2074	0.98 (0.93, 1.03)	0.475	0.98 (0.93, 1.03)	0.451	0.99 (0.94, 1.04)	0.675	0.98 (0.94, 1.02)	0.327	0.98 (0.94, 1.02)	0.251	0.97 (0.92, 1.01)	0.174	0.98 (0.94, 1.02)	0.312

The APOE *ε4* genotypes were coded as an ordered category based on the number of *ε4* possessed. Thus, level 0 encompassed *ε2ε2, ε2ε3, ε2ε3*; level 1 *ε2ε4* and *ε3 ε4*; and level 2 *ε4ε4*. Given the existence of three levels, generalized linear models with gamma distribution and orthogonal polynomial contrasts with 2 equally spaced levels (i.e., linear and quadratic) were employed to look for a dose response by *ε4* variants.

Model 2 was not adjusted for age in NSHD as all participants are age-matched.

Abbreviations as in **Supplementary Tables S1/S2**.

Supplementary Table S4. Associations between *APOE ε4* genotypes and echocardiographic data at 60-64 years by comparing non-*APOE ε4* (*ε2ε2, ε2ε3, ε2ε3*) with heterozygous-*APOE ε4* (*ε2ε4* and *ε3ε4*) and homozygous-*APOE ε4* (*ε4ε4*) genotypes.

			**M1** (unadjusted)			**M2** (adjusted for age, sex and SEP)		**M3** (M2 + BMI)		**M4** (M2 + CVD)		**M5** (M2 + diabetes)		**M6** (M2 + high cholesterol)		**M7** (M2 + HT)	
**Outcome**	**Cohort**	**Analysis**	**n**	**Exp *β* (95% CI)**	***p*-value**	**Exp *β* (95% CI)**	***p*-value**	**Exp *β* (95% CI)**	***p*-value**	**Exp *β* (95% CI)**	***p*-value**	**Exp *β* (95% CI)**	***p*-value**	**Exp *β* (95% CI)**	***p*-value**	**Exp *β* (95% CI)**	***p*-value**
**MCF**	**SABRE**	Heterozygous-*APOE ε4*	1129	1.03 (1.00, 1.07)	0.089	1.03 (1.00, 1.07)	0.086	1.03 (1.00, 1.07)	0.079	1.03 (0.99, 1.06)	0.139	1.02 (0.99, 1.06)	0.215	1.03 (0.99, 1.08)	0.201	1.03 (0.99, 1.07)	0.121
	**NSHD**	Heterozygous-*APOE ε4*	890	1.05 (1.00, 1.12)	0.070	1.06 (1.00, 1.12)	0.053	1.05 (0.99, 1.11)	0.104	1.05 (0.99, 1.12)	0.129	1.06 (1.00, 1.13)	**0.049**	1.08 (1.02, 1.15)	**0.015**	1.05 (0.99, 1.11)	0.098
	Both	Meta-analysis	2019	1.04 (1.01, 1.07)	**0.016**	1.04 (1.01, 1.07)	**0.013**	1.04 (1.01, 1.07)	**0.018**	1.03 (1.00, 1.06)	**0.043**	1.03 (1.00, 1.07)	0.060	1.05 (1.00, 1.10)	**0.040**	1.03 (1.00, 1.06)	**0.028**
	**SABRE**	Homozygous-*APOE ε4*	865	1.01 (0.92, 1.11)	0.901	1.01 (0.92, 1.11)	0.882	1.02 (0.92, 1.12)	0.746	1.00 (0.91, 1.11)	0.971	1.00 (0.91, 1.10)	0.980	1.01 (0.90, 1.14)	0.883	1.00 (0.91, 1.11)	0.967
	**NSHD**	Homozygous-*APOE ε4*	674	1.07 (0.93, 1.25)	0.353	1.08 (0.93, 1.25)	0.336	1.09 (0.94, 1.26)	0.234	1.06 (0.90, 1.26)	0.485	1.02 (0.87, 1.21)	0.786	1.02 (0.87, 1.20)	0.842	1.06 (0.92, 1.23)	0.444
	Both	Meta-analysis	1539	1.03 (0.95, 1.11)	0.544	1.03 (0.95, 1.11)	0.517	1.04 (0.96, 1.13)	0.350	1.02 (0.92, 1.10)	0.704	1.01 (0.93, 1.09)	0.874	1.01 (0.92, 1.11)	0.812	1.02 (0.94, 1.10)	0.652

All reported analyses here consisted of generalized linear models with gamma distribution and log link. Significant *p*-values are highlighted in bold.
Model 2 was not adjusted for age in NSHD as all participants are age-matched.
Abbreviations as in **Supplementary Tables S1/S2**.

Supplementary Table S5. Associations between *APOE ε4* genotypes and stroke volume data by comparing non-*APOE ε4* (*ε2ε2, ε2ε3, ε2ε3*) with any *APOE ε4* (*ε2ε4, ε3ε4 and ε4ε4*) genotypes in all cohorts.

**Outcome: Stroke Volume**		**M1** (unadjusted)			**M2** (adjusted for age, sex and SEP)		**M3** (M2 + BMI)		**M4** (M2 + CVD)		**M5** (M2 + diabetes)		**M6** (M2 + high cholesterol)		**M7** (M2 + HT)	
**Cohort**	**APOE *ε4* status**	**n**	**Exp *β* (95% CI)**	***p*-value**	**Exp *β* (95% CI)**	***p*-value**	**Exp *β* (95% CI)**	***p*-value**	**Exp *β* (95% CI)**	***p*-value**	**Exp *β* (95% CI)**	***p*-value**	**Exp *β* (95% CI)**	***p*-value**	**Exp *β* (95% CI)**	***p*-value**
**ALSPAC**	No *APOE ε4*	ref	ref	ref	ref	ref	ref	ref	ref	ref	ref	ref	ref	ref	ref	ref
	APOE ε4 carriers	1333	0.99 (0.95, 1.04)	0.756	1.00 (0.95, 1.05)	0.936	0.99 (0.95, 1.03)	0.613	0.99 (0.95, 1.04)	0.747	0,99 (0.95, 1.04)	0.953	0.98 (0.93, 1.03)	0.442	0.99 (0.94, 1.04)	0.616
**NSHD**	No *APOE ε4*	ref	ref	ref	ref	ref	ref	ref	ref	ref	ref	ref	ref	ref	ref	ref
	APOE ε4 carriers	1229	1.08 (1.03, 1.12)	**0.001**	1.06 (1.02, 1.10)	**0.006**	1.08 (1.03, 1.12)	**0.001**	1.07 (1.02, 1.12)	**0.007**	1.08 (1.04, 1.13)	**0.0006**	1.07 (1.02, 1.12)	**0.008**	1.08 (1.03, 1.12)	**0.001**
**SABRE**	No *APOE ε4*	ref	ref	ref	ref	ref	ref	ref	ref	ref	ref	ref	ref	ref	ref	ref
	APOE ε4 carriers	1160	1.02 (0.99, 1.05)	0.211	1.02 (0.99, 1.05)	0.148	1.02 (1.00, 1.05)	0.090	1.02 (0.99, 1.05)	0.248	1.02 (0.99, 1.05)	0.306	1.02 (0.99, 1.06)	0.228	1.02 (0.99, 1.05)	0.233
**UK Biobank**	No *APOE ε4*	ref	ref	ref	ref	ref	ref	ref	ref	ref	ref	ref	ref	ref	ref	ref
	APOE ε4 carriers	32644	1.00 (1.00, 1.01)	0.220	1.00 (1.00, 1.01)	0.068	1.01 (1.00, 1.01)	0.060	1.00 (1.00, 1.01)	0.275	1.00 (1.00, 1.01)	0.230	1.00 (1.00, 1.01)	0.233	1,00 (1.00, 1.01)	0.185

All reported analyses here consisted of generalized linear models with gamma distribution and log link. Significant *p*-values are highlighted in bold.
Model 2 was not adjusted for age in NSHD as all participants are age-matched.
Abbreviations as in **Supplementary Tables S1/S2**.
